# Guanabenz Reverses a Key Behavioral Change Caused by Latent Toxoplasmosis in Mice by Reducing Neuroinflammation

**DOI:** 10.1128/mBio.00381-19

**Published:** 2019-04-30

**Authors:** Jennifer Martynowicz, Leonardo Augusto, Ronald C. Wek, Stephen L. Boehm, William J. Sullivan

**Affiliations:** a Department of Microbiology and Immunology, Indiana University School of Medicine, Indianapolis, Indiana, USA; b Department of Pharmacology and Toxicology, Indiana University School of Medicine, Indianapolis, Indiana, USA; c Department of Biochemistry and Molecular Biology, Indiana University School of Medicine, Indianapolis, Indiana, USA; d Department of Psychology, Indiana University-Purdue University Indianapolis, Indianapolis, Indiana, USA; Stanford University

**Keywords:** *Toxoplasma*, behavior modification, guanabenz, host-pathogen interactions, neuroinflammation, parasites

## Abstract

Toxoplasma gondii is an intracellular parasite that has infected one-third of humans. The infection is permanent because the replicative form (tachyzoite) converts into a latent tissue cyst form (bradyzoite) that evades host immunity and is impervious to current drugs. The continued presence of these parasitic cysts hinders treatment and leads to chronic infection that has been linked to behavioral changes in rodents and neurological disease in humans. How these behavioral changes occur, and whether they are due to parasite manipulation or the host response to infection, remains an outstanding question. We previously showed that guanabenz possesses antiparasitic activity; here, we show that guanabenz reproducibly lowers brain cyst burden up to 80% in chronically infected male and female BALB/cJ mice when given intraperitoneally but not when administered by gavage or in food. Regardless of the administration route, guanabenz reverses *Toxoplasma*-induced hyperactivity in latently infected mice. In contrast, guanabenz increases cyst burden when given to chronically infected C57BL/6J mice yet still reverses *Toxoplasma*-induced hyperactivity. Examination of the brains from chronically infected BALB/cJ and C57BL/6J mice shows that guanabenz decreases inflammation and perivascular cuffing in each strain. Our study establishes a robust model for cyst reduction in BALB/cJ mice and shows for the first time that it is possible to reverse a key behavioral change associated with latent toxoplasmosis. The rescue from parasite-induced hyperactivity correlates with a decrease in neuroinflammation rather than reduced cyst counts, suggesting that some behavioral changes arise from host responses to infection.

## Abstract (precis)

Toxoplasma gondii is a common parasite of animals, including up to one-third of humans. The single-celled parasite persists within hosts for the duration of their life as tissue cysts, giving rise to chronic infection. Latent toxoplasmosis is correlated with neurological dysfunction in humans and results in dramatic behavioral changes in rodents. When infected, mice and rats adapt behaviors that make them more likely to be devoured by cats, the only host that supports the sexual stage of the parasite. In this study, we establish a new mouse model of tissue cyst depletion using a drug called guanabenz and show that it is possible to reverse a key behavior change back to normal in infected animals. We also show that the mechanism appears to have nothing to do with parasite cyst burden but rather the degree of neuroinflammation produced by chronic infection.

## INTRODUCTION

Toxoplasma gondii is a protozoan parasite in the phylum Apicomplexa that permanently infects warm-blooded vertebrates and is estimated to have infiltrated over 3 billion human hosts (1). Despite the remarkable range of host organisms, felines are the only species capable of supporting the sexual stage of the parasite’s life cycle. Upon infecting a host organism, the replicative stage of the parasite (tachyzoite) quickly converts into a quiescent stage (bradyzoite) that remains encysted in host tissues. Acute illness usually presents with minor symptoms that resolve within days as chronic infection is established. Tissue cysts housing bradyzoites are found throughout the host but primarily accumulate in large numbers in neurons of the central nervous system (CNS) (2). Bradyzoites can revert to tachyzoites during immune suppression, causing life-threatening episodes of reactivated toxoplasmosis.

Chronic infection in rodents leads to an array of CNS dysfunction, including alterations in neurotransmitter levels, protein expression, immune responses, and behavior (3–5). Behavioral changes include hyperactivity, cognitive deficits, and altered anxiety and fear responses (6–8). These behavioral changes conspire to form the basis of a popular hypothesis proposing that *Toxoplasma* manipulates its rodent hosts to become easy prey for cats (9). By driving rodent hosts into the gut of feline predators, *Toxoplasma* secures progression to the sexual stage of its life cycle and widespread transmission as oocysts in cat feces. In humans, chronic toxoplasmosis has been correlated with schizophrenia, bipolar disorder, epilepsy, rage disorder, and sudden-onset psychosis (10).

How *Toxoplasma* induces changes in host behavior remains unknown. Previous efforts to mitigate the behavioral changes caused by *Toxoplasma* have been unsuccessful. Ceftriaxone treatment can restore the altered glutamate levels and neuronal morphology in chronically infected mice but failed to reverse infection-induced changes in behavior (11). Attempts to normalize acetylcholinesterase activity in the CNS of infected mice using diphenyl diselenide did not restore altered behaviors (12). Deletion of parasite proteins such as the *Toxoplasma* AaaH2 tyrosine hydroxylase, which was hypothesized to manipulate host dopamine levels, did not impact the parasite’s influence on host behavior (13). Additionally, mice infected with an attenuated strain incapable of cyst formation continued to display behavioral alterations even though parasites could no longer be detected in these animals (14).

A means to reliably decrease or eliminate cysts from a chronically infected animal would be invaluable in dissecting mechanisms underlying *Toxoplasma*-induced behavioral changes. We previously found that the phosphorylation of *Toxoplasma* eukaryotic initiation factor 2α (TgIF2α) accompanies the stress-induced conversation of tachyzoites into bradyzoites (15, 16). As in other eukaryotic cells, phosphorylated eIF2α initiates the integrated stress response (ISR), which leads to preferential translation of mRNAs that coordinate adaptive changes to stressful stimuli (17). Guanabenz (GA), an alpha-adrenergic receptor agonist approved to treat hypertension, was recently shown to also inhibit dephosphorylation of eIF2α (18). After showing that guanabenz inhibits dephosphorylation of TgIF2α, we demonstrated that it curtails growth of tachyzoites *in vitro* and prevents reactivation of *in vitro*-generated bradyzoites (19). In the presence of guanabenz, *in vitro*-generated bradyzoites exhibited gross changes in morphology, suggesting the drug may be cysticidal (19, 20). We subsequently showed that guanabenz is active against both acute and chronic toxoplasmosis in female BALB/c mice, reducing brain cyst counts by ∼75% in those treated for latent toxoplasmosis (20).

Here, we examine the effects of guanabenz on cyst burden using different dosing and delivery strategies and an additional mouse strain, C57BL/6. We established a new mouse model in which brain cyst burden is reliably reduced by 70 to 80% with guanabenz treatment and use this model to test the hypothesis that a reduction in parasitic cysts would reverse a key behavioral change associated with chronic toxoplasmosis. Surprisingly, we found that hyperactivity in chronically infected mice can be reversed by guanabenz, but the restoration of normal locomotor activity correlates with decreased neuroinflammation rather than brain cyst burden. Importantly, this finding supports the idea that, at least for some behaviors, what appears to be host manipulation is due to the host immune response rather than a presumed neuromodulatory effector produced by the parasite.

## RESULTS

### Intraperitoneal guanabenz reduces brain cyst burden in male and female BALB/cJ mice.

**FIG 1:**
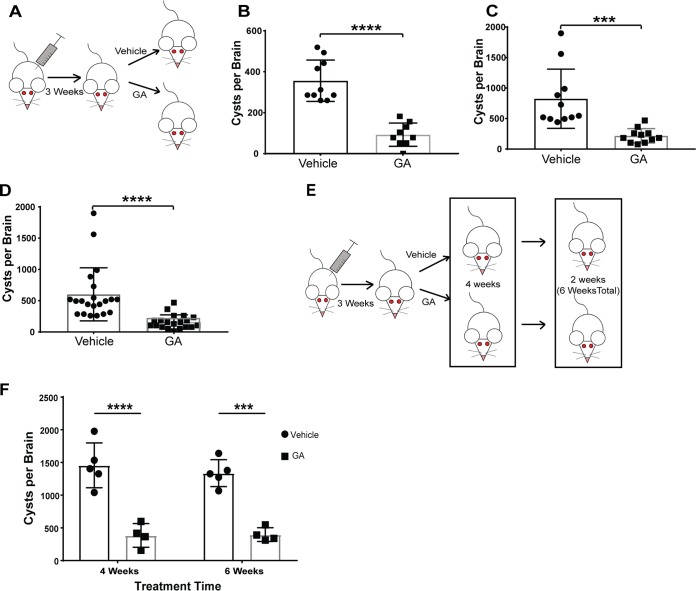
Effect of guanabenz on brain cyst burden in male and female BALB/cJ mice. (A) Female and male BALB/cJ mice were infected intraperitoneally with 10^4^ Pru tachyzoites. After 21 days, mice were randomized into either vehicle or guanabenz (GA) treatment (5 mg/kg/day i.p.). Mice were dosed for an additional 3 weeks before brains were extracted and cysts were quantified. (B and C) Males (B) and females (C) showed comparable decreases in cyst burden. Results are representative of two independent experiments. Error bars indicate standard deviation of the mean. *P* value determined using an unpaired two-tailed Student *t* test: ***, *P* < 0.001; ****, *P* < 0.0001. (D) Results shown are data from both sexes combined. Error bars indicate standard deviation of the mean. *P* value determined using an unpaired two-tailed Student *t* test: ****, *P* < 0.0001. (E) Female BALB/cJ mice were infected intraperitoneally with 10^4^ Pru tachyzoites and then randomized into either vehicle or GA (5 mg/kg/day i.p.) treatment groups. Drug treatment was initiated after establishment of chronic infection at 21 days. After 4 weeks of treatment, half of each group was examined for the 4-week time point. The remaining mice were treated for another 2 weeks for a total of 6 weeks before being examined. (F) Brain cyst counts following treatment with GA for 4 and 6 weeks. Results are representative of two independent experiments. Error bars indicate standard deviation of the mean. *P* value determined using an unpaired, two-tailed Student *t* test: ***, *P* < 0.001; ****, *P* < 0.0001.

We previously reported that guanabenz treatment reduces brain cyst burden in female BALB/c mice by 69 to 78% (20). We sought to examine whether this effect is gender specific by infecting both male and female BALB/cJ mice intraperitoneally (i.p.) with type II (Pru) parasites. Infection was allowed to progress to the chronic stage for 3 weeks before treatment with either guanabenz (5 mg/kg of body weight/day) or vehicle i.p. for 3 weeks (Fig. 1A). Brain cyst burden was quantified in the drug-treated mice and compared to the vehicle control. Our findings establish that guanabenz treatment yields an ∼75% reduction of latent brain cysts in both male and female BALB/cJ mice (Fig. 1B and C). A 73% reduction of brain cysts is observed when data from both sexes are combined (Fig. 1D). We conclude that guanabenz reproducibly lowers brain cyst burden in BALB/c mice. As the effect is not gender specific, we used only female mice in subsequent experiments.

Our lab has previously established that increasing the dosage of guanabenz to 10 mg/kg does not further reduce cyst burden (20). We next examined whether extending guanabenz treatment from 3 weeks to 4 or 6 weeks could reduce cyst counts further (Fig. 1E). Cyst counts remained reduced for the extended drug treatment times, but no further reduction was observed (Fig. 1F). It should be noted that we did not perform measurements of tachyzoites in the brain. Despite the mice being asymptomatic, low levels of tachyzoites may have been present in the mice that gave rise to the residual brain cysts that the drug treatment failed to clear. We conclude that guanabenz treatment results in an impressive and reliable decrease in cyst numbers but it does not fully eradicate the infection.

### Intraperitoneal guanabenz restores normal locomotor activity levels in chronically infected BALB/cJ mice.

**FIG 2:**
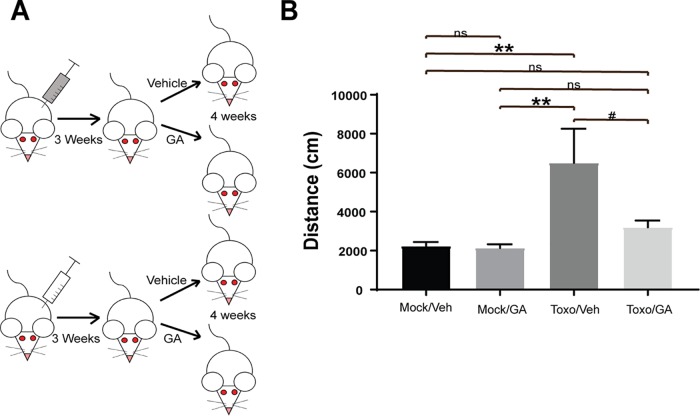
Guanabenz eliminates *Toxoplasma*-induced hyperactivity in BALB/cJ mice. (A) One group of BALB/cJ mice was infected with 10^4^ Pru tachyzoites i.p. (Toxo, gray syringe), and the other was mock infected with PBS (Mock, white syringe). Following establishment of chronic infection at 21 days, both groups were randomized into vehicle or drug (GA 5 mg/kg/day i.p.) treatment groups. (B) After 4 weeks of treatment, locomotor activity was recorded in an unfamiliar 40-cm by 4-cm by 30-cm Plexiglas box, and the total distances traveled over 30 min were compared. Error bars indicate standard error of the mean. One-way ANOVA, *P* = 0.0019, followed by an unpaired, two-tailed Student *t* test: **, *P* < 0.001; #, *P* < 0.1; ns, *P* > 0.1.

Hyperactivity is a well-established behavioral change that occurs in mice with latent toxoplasmosis (13, 21). We reasoned that since guanabenz could reduce cyst counts upward of 80%, then parasite-induced behaviors may also be attenuated. To minimize potential effects of guanabenz itself on behavior, assessment of locomotor activity was performed in the 4-h window before daily drug dosing, when there is no detectable drug in circulation or brain (22). Locomotor activity was recorded as total distance traveled (cm) in an open field over 30 min. As shown in Fig. 2A, chronically infected or mock-infected BALB/cJ mice were treated with guanabenz or vehicle daily for 4 weeks. We observed no change in locomotor activity in mock-infected mice given guanabenz, and the *Toxoplasma*-infected mice displayed the expected hyperactivity (Fig. 2B). Strikingly, the group of chronically infected mice treated with guanabenz exhibited significantly reduced hyperactivity compared to vehicle-treated mice, nearly reverting to the baseline recorded for uninfected mice (Fig. 2B).

### Oral guanabenz reduces hyperactivity without lowering cyst burden in BALB/cJ mice.

**FIG 3:**
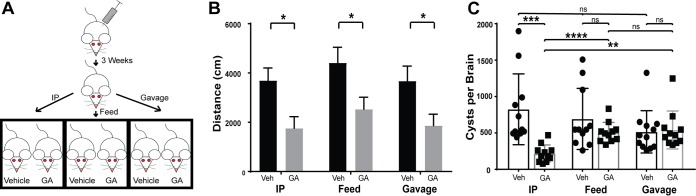
Reduction in *Toxoplasma*-induced hyperactivity is independent of cyst burden. (A) Female BALB/cJ mice were infected i.p. with 10^4^ Pru tachyzoites. Following establishment of chronic infection at 21 days, mice were randomized into groups and given either guanabenz (GA) or vehicle by different routes (i.p., feed, or gavage) for 3 weeks. (B) After 3 weeks of GA treatment, locomotor activity was recorded. Error bars indicate standard error of the mean. Single replicate with one-way ANOVA, *P* = 0.0022, followed by unpaired, two-tailed Student’s *t* test: *, *P* < 0.05. (C) After 3 weeks of GA treatment, samples were prepared in a blind manner and brain cysts were enumerated. Error bars indicate standard deviation of the mean. Single replicate with one-way ANOVA, *P* = 0.0012, followed by an unpaired, two-tailed Student *t* test: ns, *P* > 0.5; **, *P* < 0.01; ***, *P* < 0.001; ****, *P* < 0.0001.

We next examined how administration of guanabenz through oral routes compared to efficacy of i.p. drug injection. Female BALB/cJ mice were infected and allowed to progress to chronic infection as before and then were randomized into groups. Guanabenz or vehicle was administered by i.p. injection, by oral gavage, or in feed for 3 weeks before locomotor activity was recorded (Fig. 3A). Guanabenz again reversed parasite-induced hyperactivity to near-baseline levels, regardless of the dosing route used (Fig. 3B). We also quantified the brain cyst burden in these mice, again seeing a dramatic reduction in those treated with i.p. guanabenz. Surprisingly, cyst counts were not significantly altered in the latently infected mice that received guanabenz by intragastric or oral routes, suggesting that rescue from parasite-induced hyperactivity occurs independently of cyst burden (Fig. 3C).

### Guanabenz reduces chronic inflammation in infected BALB/cJ mice.

**FIG 4:**
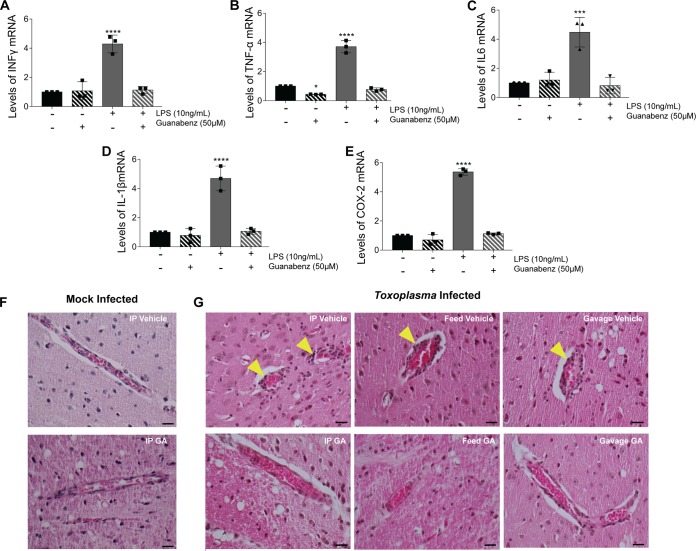
Guanabenz reduces inflammation in BALB/cJ mice *in vitro* and *in vivo*. (A to E) J774.1 cells were stimulated with LPS (10 ng/ml) in the presence or absence of guanabenz (50 µM) for 6 h. Levels of (A) IFN-γ, (B) TNF-α, (C) IL-6, (D) IL-1β and (E) COX-2 mRNA were then measured by RT-qPCR. Values of infected cells were normalized to untreated cells. Error bars indicate standard deviation of the mean (*n* = 3); Student’s *t* test: *, *P* < 0.05; ***, *P* < 0.005; ****, *P* < 0.0001. (F and G) Representative images of H&E-stained brain sections from (F) mock-infected and (G) *Toxoplasma*-infected BALB/cJ mice treated with vehicle or GA as indicated. Bar, 20 μm. Yellow arrowheads highlight the perivascular cuffing associated with chronic *Toxoplasma* infection, which is not as pronounced in GA-treated mice.

As our previous experiment showed that reduced brain cyst burden is not linked to the reversal of hyperactivity, we examined other aspects of neurophysiology that may explain the effect of guanabenz. It is well established that guanabenz exhibits anti-inflammatory properties *in vitro* and *in vivo* (22–24). *In vitro*, guanabenz has been shown to decrease the expression of proinflammatory cytokines, including IFN-γ, IL-6, and TNF-α (23, 24). Using RT-qPCR, we determined the mRNA expression levels of these inflammatory cytokines in J774.1 cells, a BALB/c-derived macrophage line (Fig. 4A to E). Stimulation of these cells using LPS resulted in increased expression of IFN-γ, TNF-α, IL-6, IL-1β, and COX-2; however, guanabenz blocked this effect. These results are consistent with the abovementioned studies showing that guanabenz has immunomodulatory properties.

We also performed histological examinations of brain sections from BALB/cJ mice from our intraperitoneal, intragastric, and oral guanabenz studies above using hematoxylin and eosin (H&E) staining. In mock-infected control mice, we found that guanabenz had no effect on baseline inflammation (Fig. 4F). Consistent with findings reported by others (5), we observed increased neuroinflammation in mice with chronic toxoplasmosis (Fig. 4G). However, chronically infected mice treated with guanabenz consistently showed reduced neuroinflammation, especially the perivascular cuffing characteristic of chronic infection, regardless of the drug delivery method used (Fig. 4G).

**FIG 5:**
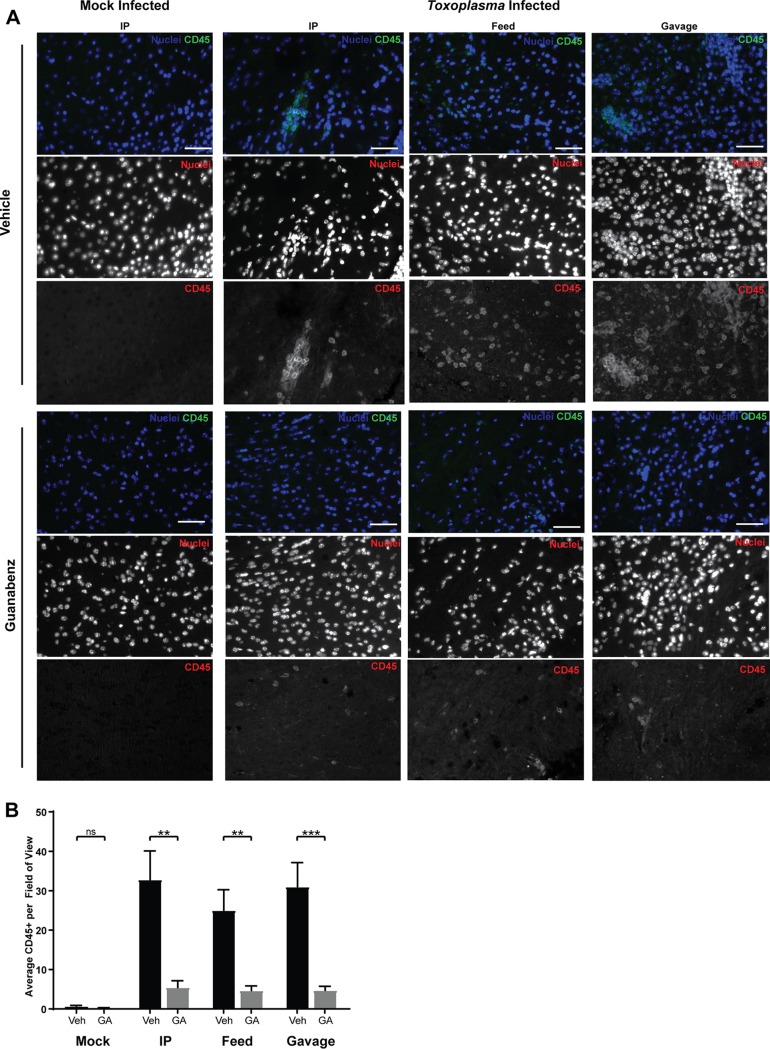
Guanabenz reduces the number of CD45^+^ cells in the brains of chronically infected BALB/cJ mice. (A) Brain tissue sections were stained with anti-CD45 antibody (green). Hoechst stain was used to label nuclei (blue). Representative images for each group are shown. (B) Five random fields of view were selected under Hoechst staining for each mouse. Images were then randomized and prepared in a blind manner before the number of CD45^+^ cells was counted. Average number of CD45^+^ cells per field for each mouse was calculated and visualized. Error bars indicate standard deviation of the mean. Bar, 50 μm. Single replicate with one-way ANOVA, *P* < 0.0001, followed by an unpaired, two-tailed Student *t* test: ns, *P* > 0.5; **, *P* < 0.01; ***, *P* < 0.001.

To quantify the reduction in neuroinflammation, we used immunohistochemistry (IHC) to label sections of brain using anti-CD45 antibody, which serves as a general marker for immune cells by identifying leukocytes (Fig. 5A). Regardless of the administration route, guanabenz caused a reduction in CD45^+^ cells in chronically infected BALB/cJ mice (Fig. 5B). No difference in CD45^+^ cells was detected in the brains from mock-infected mice. Considered with the H&E data, our results show that the reversal of hyperactivity seen in chronically infected mice treated with guanabenz is driven by reduced neuroinflammation rather than parasite cyst burden. These findings imply that some behavior changes induced by latent toxoplasmosis are more a consequence of chronic neuroinflammation rather than a parasite-derived effector.

### Guanabenz is detrimental to survival of chronically infected C57BL/6J mice.

To further examine the mechanism of guanabenz against *Toxoplasma in vivo*, we performed a series of similar experiments in C57BL/6J mice to determine if the effects of guanabenz were the same in an independent, nonisogenic mouse strain. Moreover, C57BL/6J mice are commonly employed when investigating the immune response to *Toxoplasma*. C57BL/6J mice are highly susceptible to *Toxoplasma* infection, displaying enhanced pathology and higher cyst burdens than more resilient strains like BALB/cJ (25, 26).

**FIG 6:**
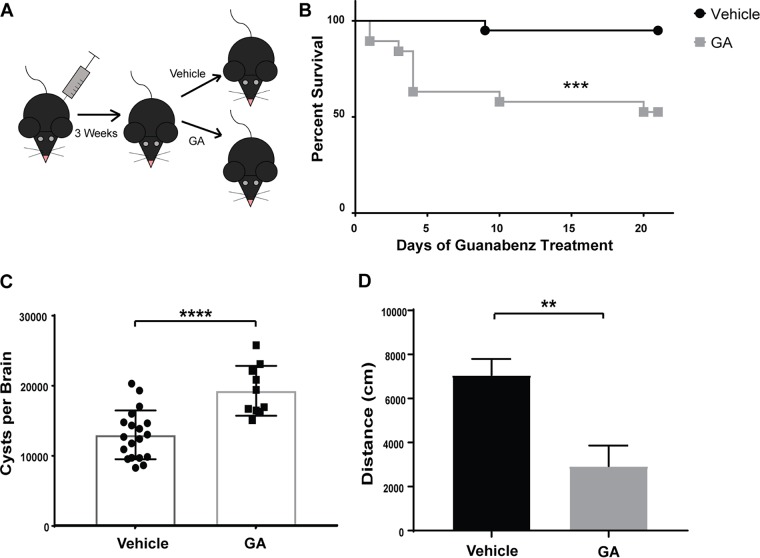
Effects of guanabenz on *Toxoplasma* cyst burden and behavior in C57BL/6 mice. (A) Male and female C57BL/6 mice were infected i.p. with 10^3^ Pru tachyzoites. Following establishment of chronic infection at 21 days, groups were randomized to receive vehicle or guanabenz (GA 5 mg/kg/day i.p.). (B) Survival of mice in designated treatment groups. Single replicate with both sexes, log rank test, *P* = 0.00232; ***, *P* < 0.001. (C) Brain cyst quantification was performed in a blind manner on the vehicle- and GA-treated C57BL/6 mice. Error bars indicate standard deviation of the mean. *P* values determined by Student’s *t* test: **, *P* < 0.01; ***, *P* < 0.001; ****, *P* < 0.0001. (D) Following 3 weeks of treatment, locomotor activity was recorded. Error bars indicate standard error of the mean. *P* values determined by Student’s *t* test: *, *P* < 0.05; **, *P* < 0.01.

Both male and female C57BL/6J mice were injected with a nonlethal dose of *Toxoplasma* to establish chronic infection for 3 weeks (Fig. 6A). Mice were then randomized into groups before being treated with either guanabenz or vehicle i.p. for 3 weeks. Unexpectedly, guanabenz caused nearly half of the chronically infected mice to die within the first 10 days of drug treatment (Fig. 6B). Mice that succumbed to the guanabenz treatment developed classic symptoms of *Toxoplasma* encephalitis, including lethargy, tremors, paralysis, and seizure. None of these mice showed signs of active infection at the start of guanabenz treatment. These results suggest that the mice experienced reactivation of encysted parasites rather than continuation of a severe acute infection, but we cannot rule out the presence of lingering tachyzoites at the start of drug treatment. The mice that survived the full drug treatment did not display any symptoms of *Toxoplasma* encephalitis. Stratifying the groups by sex shows that the effects were equal in severity among both male and female C57BL/6J mice (see Fig. S1A in the supplemental material).

10.1128/mBio.00381-19.1 FIG S1 Effects of guanabenz treatment in chronically infected male and female C57BL/6J mice. (A) Survival of C57BL/6J mice with latent toxoplasmosis following treatment with guanabenz (GA). When the sexes are split, both trend together, showing that the lethal drug effects are not sex dependent. Single replicate, log rank test, male *P* = 0.072, female *P* = 0.0128. *, *P* < 0.05; #, *P* < 0.08. (B and C) Both male (B) and female (C) mice showed an increase in cyst burden following GA treatment. (D and E) Following 3 weeks of GA treatment, locomotor activity was recorded in an unfamiliar 40-cm by 4-cm by 30-cm Plexiglas box, and the total distances traveled over 30 minutes were compared. Total distances traveled were compared across all groups. Two vehicle control mice and two GA-treated mice were excluded due to neurological deficits that impeded movement, as determined by an individual blind to treatment group. Both male (D) and female (E) mice showed a decrease in *Toxoplasma*-induced hyperactivity in the surviving mice, despite the higher cyst burden. Single replicate followed by an unpaired, two-tailed Student *t* test: ns, *P* > 0.5; *, *P* < 0.05; **, *P* < 0.01; ***, *P* < 0.001. Download FIG S1, EPS file, 2.1 MB. Copyright © 2019 Martynowicz et al. 2019 Martynowicz et al. This content is distributed under the terms of the Creative Commons Attribution 4.0 International license.

### Guanabenz increases cyst burden in C57BL/6J mice yet still reduces hyperactivity and inflammation.

**FIG 7:**
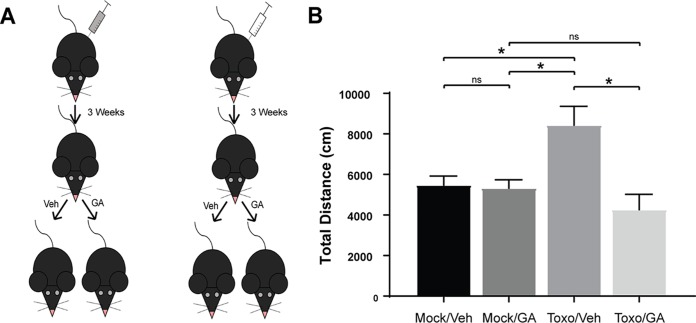
Guanabenz reverses *Toxoplasma*-induced hyperactivity, but not baseline activity, in C57BL/6J mice. (A) Female C57BL/6J mice were either mock infected with PBS (Mock, white syringe) or infected with 10^3^ Pru tachyzoites i.p. (Toxo, gray syringe) and allowed to progress to chronic infection for 21 days. Both groups were then randomized into two treatment groups (vehicle or GA 5 mg/kg/day i.p.) and treated for 3 weeks. (B) Following 3 weeks of treatment, locomotor activity was recorded. Single replicate, one-way ANOVA, *P* = 0.0024, followed by an unpaired, two-tailed Student *t* test: *, *P* < 0.05.

In contrast to its effect in BALB/cJ mice, i.p. guanabenz led to increased cyst burdens in the surviving C57BL/6J mice (Fig. 6C; Fig. S1B and C). Despite the increase in brain cyst counts in the guanabenz-treated C57BL/6 mice, the drug still reversed *Toxoplasma*-induced hyperactivity (Fig. 6D; Fig. S1D and E). The experiment was repeated with the inclusion of mock-infected mice (Fig. 7A). The decrease in hyperactivity was observed again in drug-treated chronically infected mice, but there was no effect on activity level in the mock-infected mice receiving guanabenz (Fig. 7B). These results match those observed with BALB/cJ mice.

**FIG 8:**
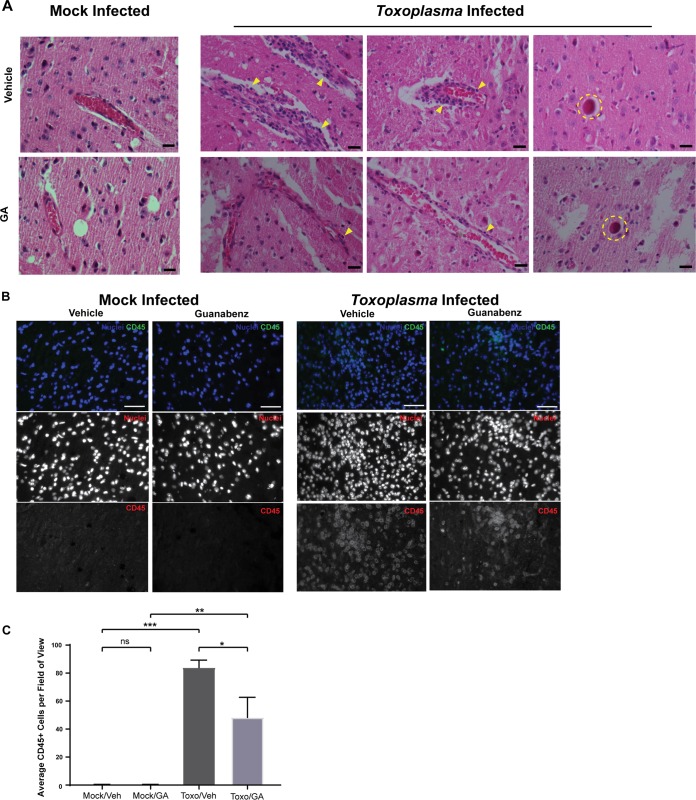
Guanabenz reduces chronic inflammation in C57BL/6J mice with latent toxoplasmosis. (A) Representative H&E-stained images of mock-infected and *Toxoplasma*-infected brains from vehicle control and GA-treated C57BL/6J mice. Staining patterns indicate a decrease in chronic inflammation and perivascular cuffing (yellow arrowheads). There is also a lack of immune presence around tissue cysts (dashed yellow circle) in each group, which is typical of chronic *Toxoplasma* infection. Bar, 20 μm. (B) Representative brain tissue sections stained with anti-CD45 antibody (green) and Hoechst stain (blue) for designated group. Bar, 50 μm. (C) Five random fields of view were selected under Hoechst staining for each mouse. Images were then randomized and prepared in a blind manner before the number of CD45^+^ cells was counted. Average number of CD45^+^ cells per field for each mouse was calculated and visualized. Error bars indicate standard error of the mean. Single replicate with one-way ANOVA, *P* < 0.0001, followed by an unpaired, two-tailed Student *t* test: ns, *P* > 0.5; *, *P* < 0.05; **, *P* < 0.01; ***, *P* < 0.001.

Histological examination of the brains from infected C57BL/6J mice treated with vehicle showed the overwhelming level of inflammation that is characteristic of latent toxoplasmosis in this mouse strain (Fig. 8A). As previously reported (5), this inflammation does not localize to tissue cysts but remains concentrated around the vasculature and diffuse throughout the cortex. When the brains were examined using IHC with anti-CD45 antibody, the increased inflammation following *Toxoplasma* infection was confirmed (Fig. 8B). Quantification of this inflammation shows a statistically lower number of CD45^+^ cells in the guanabenz-treated mice (Fig. 8C). These findings bolster the idea that reversal of the hyperactive behavior depends more on controlling neuroinflammation than cyst burden.

## DISCUSSION

A key strategy that many parasites use to facilitate transmission involves behavioral modification of its host. *Toxoplasma* induces behavioral changes in chronically infected mice and rats, which have been proposed to increase the odds that the rodent would be captured and consumed by the definitive feline host of *Toxoplasma*. These behavior changes include hyperactivity, cognitive impairment, and diminished fear of cat odors (6, 8, 27). Several hypotheses that are not mutually exclusive have emerged to explain how *Toxoplasma* may manipulate its host. *Toxoplasma* may cause structural damage to the brain that affects neurobiological processes, but to date there is no consensus on the parasite’s distribution within the brain (5, 8, 28). *Toxoplasma* may release parasite effector proteins that lead to altered levels of neurotransmitters or hormones in the host, but attempts to restore neurotransmitter levels through pharmacological intervention thus far have failed to reverse behavioral changes (11, 12). Finally, behavioral changes could be an indirect consequence of the host immune response, such as neuroinflammation (4, 29). The findings we present in this study lend support to this third possibility.

We sought to determine if behavioral changes correlate with brain cyst burden by taking advantage of guanabenz, an FDA-approved drug for hypertension that was recently shown to also have activity against apicomplexan parasites (19, 20). After trying several doses and various routes of administration, and checking for different responses between sexes, we optimized conditions that reproducibly lower brain cyst burden by 70 to 80% in BALB/cJ mice (Fig. 1). The establishment of this mouse model for brain cyst reduction should prove invaluable in the future study of questions designed to interrogate effects of latent toxoplasmosis on behavior. For this study, we chose to examine hyperactivity, as it was the most consistent and reproducible behavioral change observed in our hands. We also attempted to examine anxiety, working memory, and odor aversion, but these behavioral assays produced inconsistent results between experiments.

Three-week treatment of chronically infected BALB/cJ mice with guanabenz consistently reversed *Toxoplasma*-induced hyperactivity, leading us to believe that cyst burden correlated with this change in behavior (Fig. 2). However, the rescue from hyperactivity was also seen when guanabenz was administered through oral routes that had no impact on brain cyst counts (Fig. 3). The difference between oral and i.p. administration is likely due to slower absorption through the gut and the effects of first-pass metabolism. Consistent with this idea, there was a delay in the hypotensive effects caused by GA in the mice that were given drug by gavage. Mice given i.p. GA showed signs of hypotension (such as staggering gait) within minutes of injection, but mice given GA by gavage did not display hypotensive side effects until ∼30 min later. Importantly, treatment of chronically infected C57BL/6 mice did not lower cyst counts and yet still reduced parasite-induced hyperactivity (Fig. 6 and 7). Taken together, these results suggest that the number of brain cysts is not associated with this change in behavior.

We then addressed an alternative possibility: the behavioral changes induced by latent toxoplasmosis could arise from chronic neuroinflammation. Indeed, guanabenz has previously been shown to have anti-inflammatory properties and is currently in clinical trials for multiple sclerosis (22–24). To test this idea, we examined brain tissue harvested from guanabenz-treated or vehicle-treated mice that were harboring latent toxoplasmosis. In addition to H&E staining, we used anti-CD45 staining to quantify the degree of immune presence in the brain (Fig. 4 and 5). Consistent with other reports (5, 11), the latently infected brains in our study continued to show inflammation despite the absence of acute toxoplasmosis symptoms. Guanabenz reduced neuroinflammation in mice suffering from chronic infection and was the common feature observed in any mouse that exhibited a reversal of hyperactivity to baseline levels. Our data show that the restoration of *Toxoplasma*-induced hyperactivity correlates with reduced neuroinflammation rather than brain cyst burden. Dissecting the nature of this chronic inflammatory response should reveal better insights into the mechanisms underlying the behavioral changes observed during latent toxoplasmosis.

Neuroinflammation has been repeatedly suggested as a major player in global CNS dysfunction. The brain is considered an immune-privileged site with its own resident macrophage (microglia) to maintain homeostasis. Elevated levels of inflammation have been associated with behavioral changes in rodent models and have been linked with some of the neurological diseases with which *Toxoplasma* correlates most strongly in humans, such as schizophrenia (30). Schizophrenic patients given antipsychotics with anti-*Toxoplasma* activity show a decrease in symptoms along with a decrease in inflammatory markers (31). Additionally, other pathogens, including herpes simplex virus, have been proposed to be a factor in the development of neuropathological diseases, in particular Alzheimer’s disease (32).

Our findings bolster the idea that neuroinflammatory mechanisms driven by *Toxoplasma* infection influence host CNS function. It has been proposed that extensive cytokine secretion and NF-κB activation induced by the parasite mediate neurobehavioral effects (33). An analysis of synaptosomal protein composition found that inflammation-related responses are upregulated during chronic toxoplasmosis in mice (4). In addition, multiple transcriptomic analyses show immune-specific transcripts to be among the most highly upregulated in chronic infection (5, 34, 35). Immune infiltration was also found to persist for weeks in mice that overcame infection with an attenuated strain of *Toxoplasma* that formed no detectable cysts *in vivo* (14).

The fact that we observed vastly different effects of guanabenz on two different strains of mice highlights that *in vivo* studies of *Toxoplasma* should be performed in multiple (nonisogenic) strains of mice before firm conclusions can be drawn. BALB/cJ and C57BL/6J exhibit important differences in their immune response to *Toxoplasma* infection, and the use of guanabenz may be helpful in future studies designed to further interrogate the molecular differences between these strains of mice in response to infection. C57BL/6J mice show greatly enhanced pathology due to differences mapped to the major histocompatibility complex (MHC) class II haplotype, which is found on antigen-presenting cells (25). When C57BL/6J mice were treated with guanabenz, almost half of the drug-treated group died after displaying signs of acute toxoplasmosis that could have arisen from reactivation of tissue cysts or lingering tachyzoites that may have been present during administration of guanabenz. We suspect that the anti-inflammatory effects, especially the guanabenz-mediated reduction in IFN-γ known to be critical in controlling pathogenesis (Fig. 4), are reducing the ability of the more susceptible host to control reactivated parasites. It is also important to emphasize that guanabenz activity differed when administered orally instead of intraperitoneally. It is likely that the slower absorption through the gut during intragastric dosing, along with first-pass metabolism effects, prevented brain cysts from being significantly affected.

Despite longer treatment times, guanabenz was unable to lower cyst burden further or eliminate cysts altogether. Curiously, similar results were seen with endochin-like quinolones, which inhibit the parasite cytochrome *bc*_1_ complex (36), and an inhibitor of Toxoplasma gondii calcium-dependent protein kinase 1 referred to as compound 32 (37). It is unclear why, in all cases, brain cyst counts could not be lowered beyond ∼80%. Future studies that monitor where these intractable populations hide in the brain may shed light on this conundrum. We did not measure tachyzoite levels in the brain, which could also have given rise to the residual tissue cysts. Cyst eradication is a vital area for continued study and has important clinical ramifications. Not only do cysts give rise to chronic infection in humans and life-threatening reactivation of acute infection in immunocompromised patients, but they have also been linked to neuropsychiatric disorders such as schizophrenia, intermittent explosive disorder, and suicide (10, 29). Aside from cyst reduction, our studies also suggest that certain neuropsychiatric disorders, if linked to latent toxoplasmosis, might be controlled through anti-inflammatory therapies.

## MATERIALS AND METHODS

### Parasite strains and culture.

Toxoplasma gondii parasites (type II Prugniaud [Pru] strain) were collected as tissue cysts from chronically infected BALB/c mice and used to infect human foreskin fibroblasts (HFF). The infected cultures were maintained in Dulbecco’s medium supplemented with 1% heat-inactivated fetal bovine serum (FBS) in a humidified incubator at 37°C with 5% CO_2_. To ensure their developmental capacity, tachyzoites were maintained in culture no more than 15 passages. Parasites were tested for mycoplasma using PCR as previously described prior to use in mice (38).

### Mouse strains and infection.

The mice used in this study were housed in American Association for Accreditation of Laboratory Animal Care (AAALAC)-approved facilities at either the Indiana University School of Medicine Laboratory Animal Research Center (LARC) or the IUPUI Science Animal Research Center (SARC). The Institutional Animal Care and Use Committee (IACUC) at Indiana University School of Medicine approved the use of all animals and procedures (IACUC protocol numbers 10852 and 11376).

BALB/cJ mice were purchased from the Jackson Laboratory (Bar Harbor, ME) at 5 weeks of age. The mice were allowed to acclimate for 1 week before being intraperitoneally infected with 10^4^ Pru tachyzoites suspended in 100 μl of autoclaved, filter-sterilized PBS. C57BL/J6 mice, also purchased from the Jackson Laboratory at 5 weeks of age, were infected with 10^3^ Pru tachyzoites after acclimation. Mice were routinely observed multiple times a day throughout the course of acute infection. Mock-infected animals were handled in an identical manner and injected with the same volume of sterile PBS. After 21 days postinfection, mice were randomized into treatment groups. Blood samples were extracted by cardiac puncture to confirm parasite infection by serological analysis using a dot blot containing parasite lysate.

### Drug administration.

All drug treatments in this study were administered after mice had progressed to the chronic stage of infection (21 days postinfection), at which point they were no longer symptomatic and had started to regain weight. Guanabenz was delivered i.p. at 5 mg/kg/day at the same time every day for 3 to 6 weeks depending on the experiment. For oral gavage, the dosage was increased to 10 mg/kg/day but administered at the same time as in the i.p. group. For the medicated feed group, fresh feed was made every other day and old feed was removed when fresh feed was added. To minimize the influence of feed buried in the cage bedding on fresh feed consumption, the cages for this group were changed every 4 days. Feed (5 g/mouse/day) was weighed out, placed in a metal bowl, combined with 5.5 ml water per g of feed, and allowed to absorb for 15 min. Separate bowls were used for the vehicle group and drug treatment group. The calculated dose of guanabenz for 10 mg/kg/day/mouse was suspended in 10 ml of corn oil (or 10 ml of corn oil alone for the vehicle control) and added to the wet feed before being mixed with a potato masher and placed in a plastic weigh boat. The weigh boat was placed on the floor of the cage where the mice could easily reach it.

### Locomotor activity.

Twenty to 24 h after the last drug treatment, mice were moved to the testing room and allowed to acclimate for 30 min. Prior to performing the locomotive assay, mice were evaluated by an individual blind to the study groups, and any mouse that exhibited signs of illness or a symptom that would limit movement (such as limb paralysis) was noted and removed from the mobility analysis. In total, two vehicle-treated mice and two guanabenz-treated mice were excluded from the experiment shown in Fig. 7. Individual mice were then placed in a VersaMax locomotor activity testing chamber (AccuScan Instruments), and locomotion was recorded for 30 min. The activity chamber measured 40 by 40 cm and was housed within a sound-attenuating chamber with a house light and fan for ventilation. The chamber was equipped with photocell beams located 2 cm above the Plexiglas floor to record locomotor activity. Mice were returned to their home cage after the 30-min test session.

### Brain cyst burden and histological analysis.

Brains were extracted and cut in half; the left hemisphere was processed for cyst quantification, and the right hemisphere was fixed in 10% neutral buffered formalin (NBF) for histology. Cyst burden was quantified as previously described (20). Briefly, the dissected tissue was homogenized in 650 μl of sterile PBS using a mortar and pestle. A 250-μl aliquot of homogenate was fixed using 3% methanol-free formaldehyde for 20 min. The homogenate was blocked using 3% bovine serum albumin (BSA) in 0.2% Triton X-100 before staining with 1:250 rhodamine-conjugated Dolichos biflorus lectin (Vector Laboratories) to visualize the cyst wall. Five-microliter aliquots of stained homogenate were placed on a coverslip and sealed before imaging. Stained samples were prepared in a blind manner before cysts were counted under ×20 magnification.

Following a week in 10% NBF, the fixed tissue was processed using dehydration through graded ethanol and xylene before being embedded into paraffin blocks. Blocks were cut into 5-μm sections using a microtome and then heat fixed to glass slides. Slides were stained with hematoxylin and eosin and then viewed on a Leica DM 2500 microscope. Representative images were taken from each treatment and infection status group, with particular focus given to the perivascular regions.

For IHC, slides were rehydrated through graded xylene and methanol before antigen retrieval using citric buffer. Sections were then blocked using 10% normal goat serum, 1% bovine serum albumin in PBS-T for 2 h at room temperature. Rat anti-mouse CD45 antibody (Stem Cell Technologies; lot no. BX30892) diluted 1:400 in blocking buffer was added for 1 h before repeat washes in PBS-T. Goat anti-rat secondary antibody (Invitrogen; Alexa Fluor 488 lot no. 1928689) diluted 1:200 in blocking buffer was added for 1 h in the dark. After serial washes with PBS‐T, sections were incubated with 20 μg/ml Hoechst 33342 in PBS‐T for 10 min to visualize nuclei. Coverslips were mounted using PermaFluor mountant medium (no. TA‐030‐FM; Thermo). Slides were viewed using a Nikon Eclipse E100080i microscope, and digital images were captured with a Hamamatsu C4742‐95 charge-coupled device camera using NIS elements software. Five random fields of view were selected and captured while viewing nuclei. Images were prepared in a blind manner and randomized before CD45^+^ cells were counted in each image. The average number of CD45^+^ cells was calculated for each mouse.

### Measurement of mRNA levels.

J774.1 cells at 1 × 10^5^ were seeded into 6-well plates and allowed to adhere overnight. Cells were stimulated with lipopolysaccharide (10 ng/ml) for 6 h in the presences or absence of guanabenz (50 μM) as previously reported (23). Total RNA was isolated from the cells using TRIzol LS (Invitrogen), and cDNA was generated using Omniscript (Qiagen). RT-qPCR was carried out using SYBR Green real-time PCR master mixes (Invitrogen) and the StepOnePlus Real system (Applied Biosystems). Relative levels of transcripts were calculated with the ΔΔ*C_T_* method using GAPDH as the internal control. The relative levels of the target mRNAs from the untreated cells were adjusted to 1 and served as the basal control value. Each experiment was performed three times, each with three technical replicates. The primers used are listed in Table S1 in the supplemental material.

10.1128/mBio.00381-19.2 TABLE S1 List of primers used for RT-qPCR. Download Table S1, DOCX file, 0.01 MB. Copyright © 2019 Martynowicz et al. 2019 Martynowicz et al. This content is distributed under the terms of the Creative Commons Attribution 4.0 International license.

### Statistics.

Statistical analysis was performed using GraphPad Prism v7.03. For data sets comparing two groups, statistical significance was determined using Student’s *t* test. If more than two groups were compared, the data sets were analyzed using one-way ANOVA to determine statistical significance before secondary analysis with Student’s *t* test. Survival data were analyzed using a log rank test. *P* values of ≤0.05 were considered statistically significant.
