# Supplementary material for: Guanabenz Reverses a Key Behavioral Change Caused by Latent Toxoplasmosis in Mice by Reducing Neuroinflammation
Source: mBio. 2019 Apr 30;10(2):e00381-19. doi: 10.1128/mBio.00381-19 (PMC6495372; doi:10.1128/mBio.00381-19)
Supplement: TABLE S1 [file mBio.00381-19-st001.docx]

**Supplemental Table 1. List of primers used for RTqPCR**

| Gene | Forward Primer | Reverse Primer |
| --- | --- | --- |
| IL-1β | 5’-GCCCATCCTCTGTGACTCAT-3’ | 5’-AGGCCACAGGTATTTTGTCG-3’ |
| IL-6 | 5’-TTCCATCCAGTTGCCTTCTT-3’ | 5’-TCCACGATTTCCCAGAGAAC-3’ |
| TNF-α | 5’-GAACTGGCAGAAGAGGCACT-3’ | 5’-AGGGTCTGGGCCATAGAACT-3’ |
| COX2 | 5’-CCCCCACAGTCAAAGACACT-3’ | 5’-CTCATCACCCCACTCAGGAT-3’ |
| GAPDH | 5’-TGCACCACCAACTGCTTAG-3’ | 5’-GGATGCAGGGATGATGTTC-3’ |
| IFN-γ | 5’-TTCTTCAGCAACAGCAAGGC-3’ | 5’-TCAGCAGCGACTCCTTTTCC-3; |
